# Supplementary material for: Human-derived NLS enhance the gene transfer efficiency of chitosan
Source: Biosci Rep. 2021 Jan 6;41(1):BSR20201026. doi: 10.1042/BSR20201026 (PMC7789810; doi:10.1042/BSR20201026)
Supplement: Supplementary Figures S1-S10 [file BSR-2020-1026_supp.pdf]

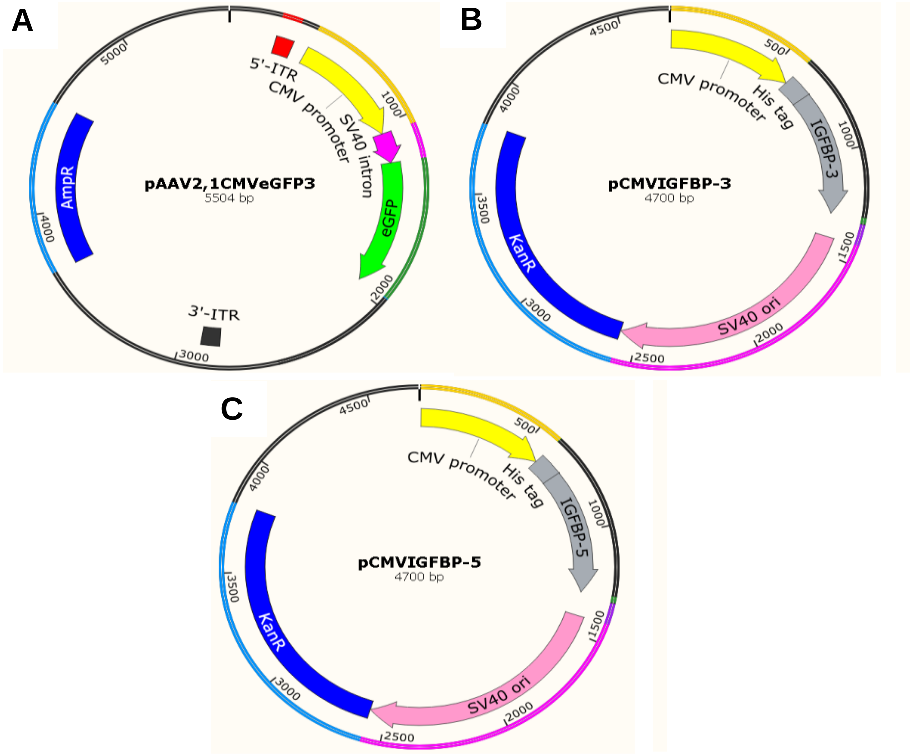

Figure S1 - **Schematic representation of the structure of plasmids.**
(A) pAAV2.1CMVeGFP3 used for expression of GFP (B) pCMVIGFBP-3 used to encode IGFBP-3 peptide and (C) pCMVIGFBP-5 used to encode IGFBP-5 peptide. AmpR and KanR are genes for resistance to ampicilin and kanamycin, respectively.

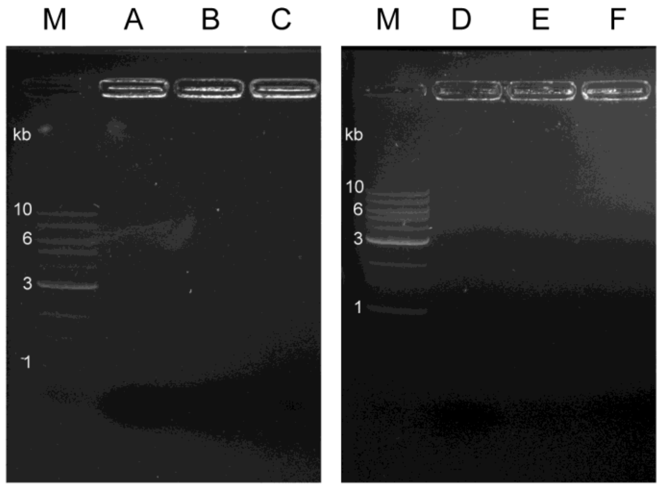

Figure S2 –Complexation assay to evaluate the polyplexes ability to complex DNA.

Representative images of the evaluation of DNA complexation by polyplexes by agarose gel electrophoresis and visualized by GreenSafe Premium. Polyplexes display efficient DNA complexation, evidenced by the absence of free DNA migration on the agarose gel. M – 1kb DNA Marker; A – CS3 (T) 100 µg; B – CS5 (T) 100 µg; C – 15:1CS3 (T); D – 15:1CS3 (S); E – 15:1CS5 (T); F – 15:1CS5 (S).

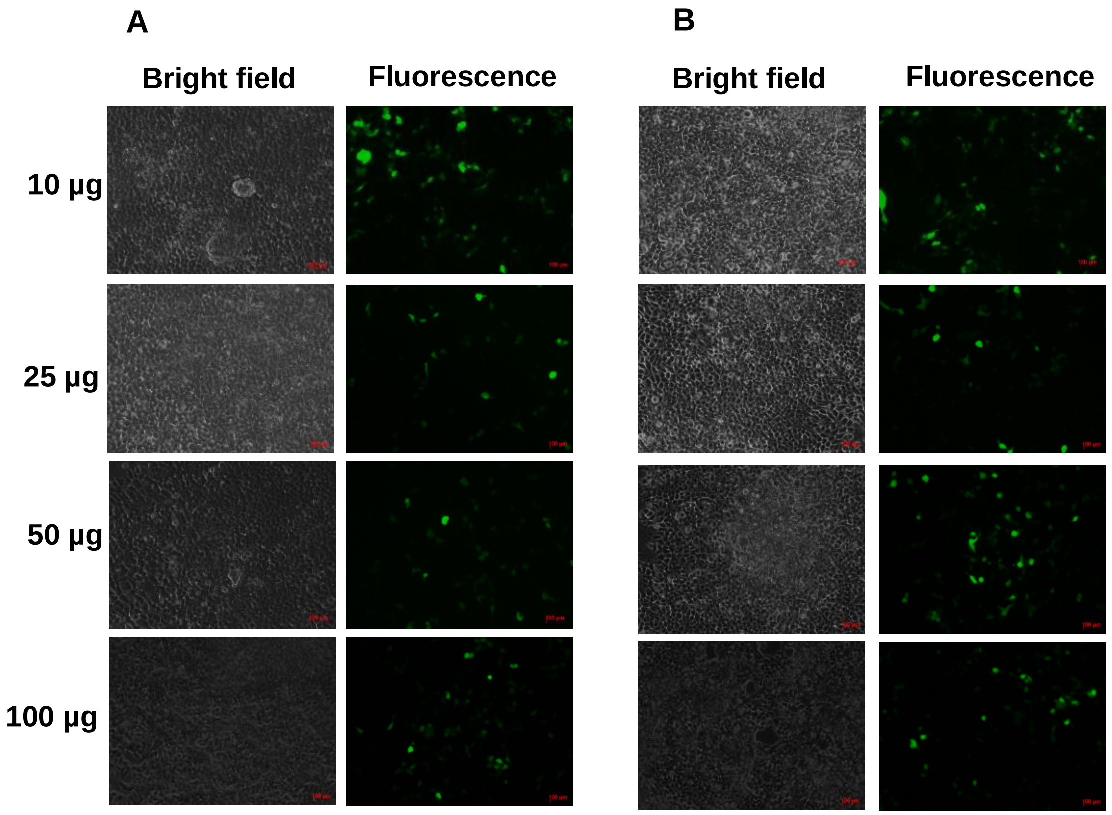

Figure S3 - **Representative images of fluorescence microscopy of transfected cells by $CSNa_2SO_4$ polyplexes**.

Transfection of $CSNa_2SO_4$ polyplexes with several concentrations of IGFBP-3, after 48h (A) and 72h (B). Amplification of 100X and scale bar represents 100 µm.

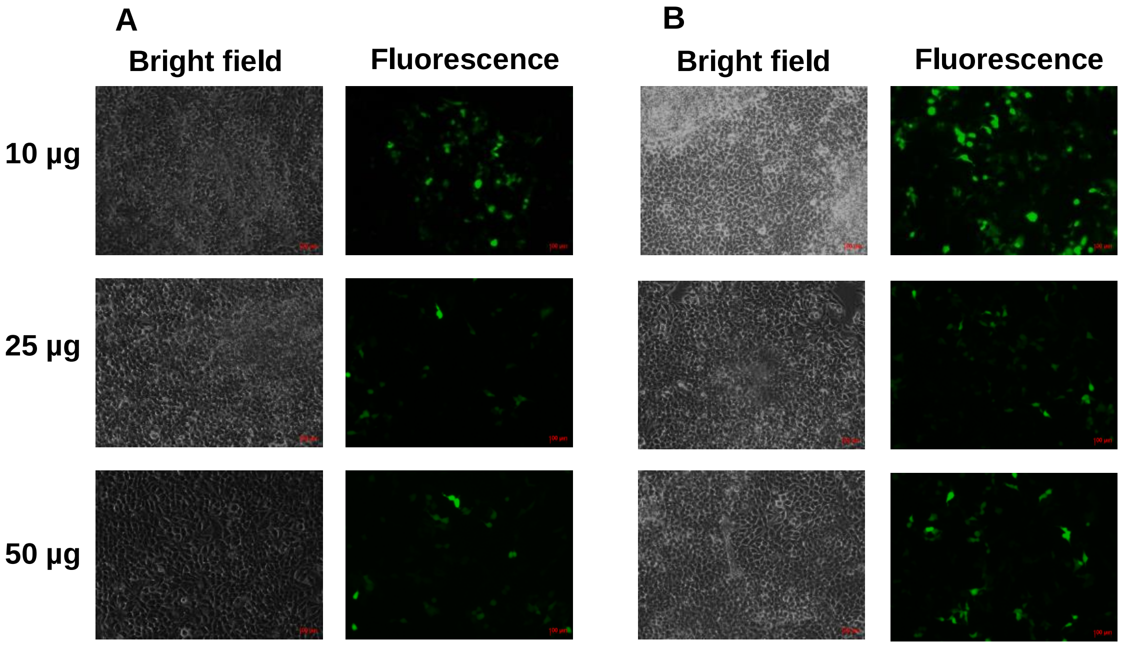

Figure S4 - **Representative images of fluorescence microscopy of transfected cells by $CSNa_2SO_45$ polyplexes**.
Transfection of $CSNa_2SO_45$ polyplexes with several concentrations of IGFBP-5 peptides, after 48h (A) and 72h (B). Amplification of 100X and scale bar represents 100 µm.

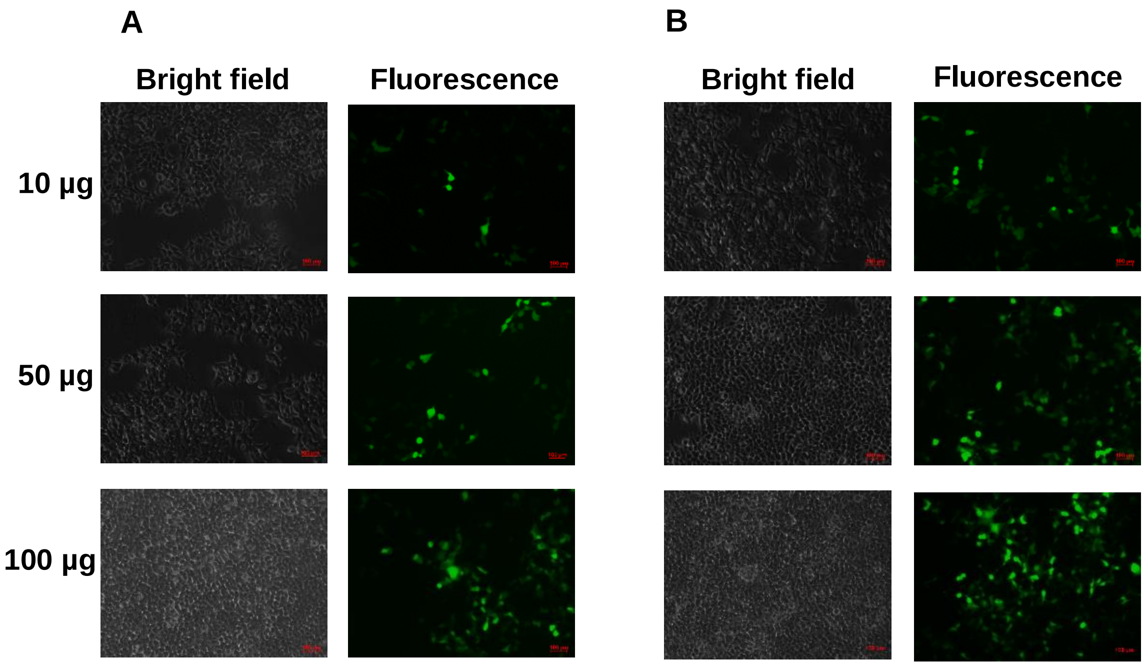

Figure S5 - **Representative images of fluorescence microscopy of transfected cells by CS3 (S) polyplexes**.
Transfection of CS3 (S) polyplexes with several concentrations of IGFBP-3, after 48h (A) and 72h (B). Amplification of 100X and scale bar represents 100 µm.

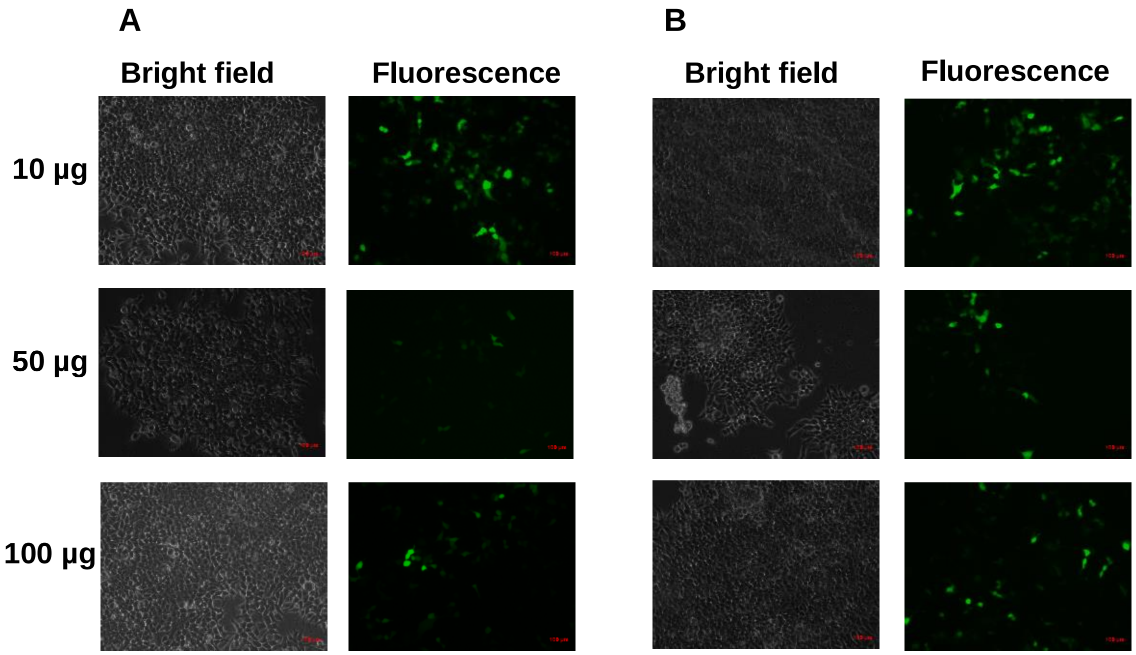

Figure S6 - **Representative images of fluorescence microscopy of transfected cells by CS5 (S) polyplexes.**

Transfection of CS5 (S) polyplexes with several concentrations of IGFBP-5, after 48h (A) and 72h (B). Amplification of 100X and scale bar represents 100 µm.

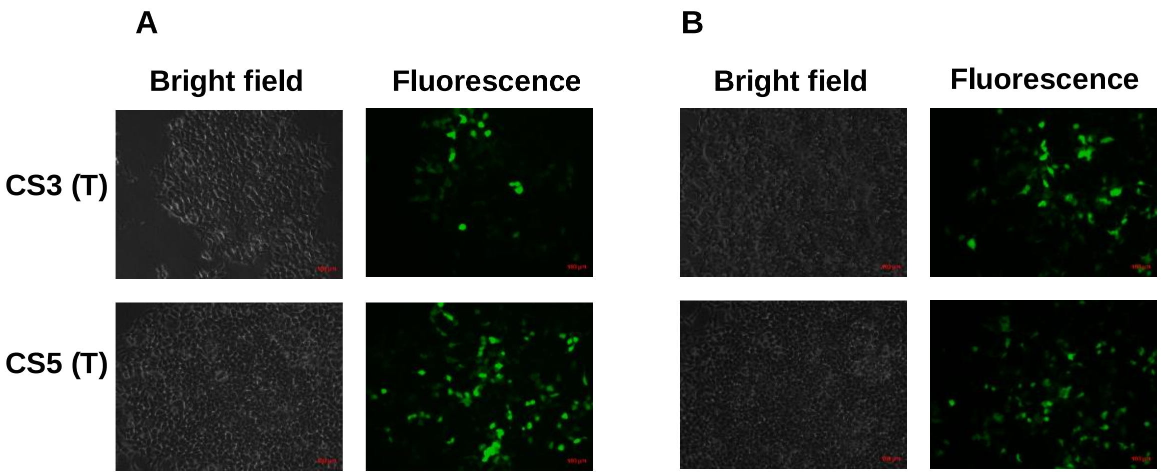

Figure S7 - **Representative images of fluorescence microscopy of transfected cells by CS3 and CS5, (T), polyplexes.**

Transfection of CS3 and CS5, (T) with 100 µg of IGFBP-3 or IGFBP-5, respectively. Cells were visualized after 48h (A) and 72h (B). Amplification of 100X and scale bar represents 100 µm.

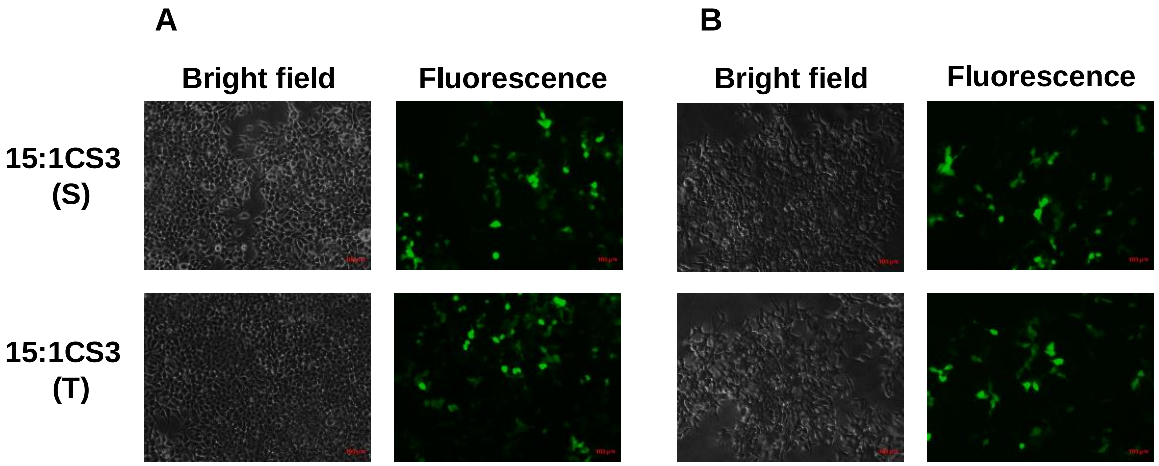

Figure S8 - **Representative images of fluorescence microscopy of transfected cells by 15:1CS3 (S) and 15:1CS3 (T) polyplexes.**

Transfection of 15:1CS3 (S) and 15:1CS3 (T) polyplexes with 100 µg of IGFBP-3, after 48h (A) and 72h (B). Amplification of 100X and scale bar represents 100 µm.

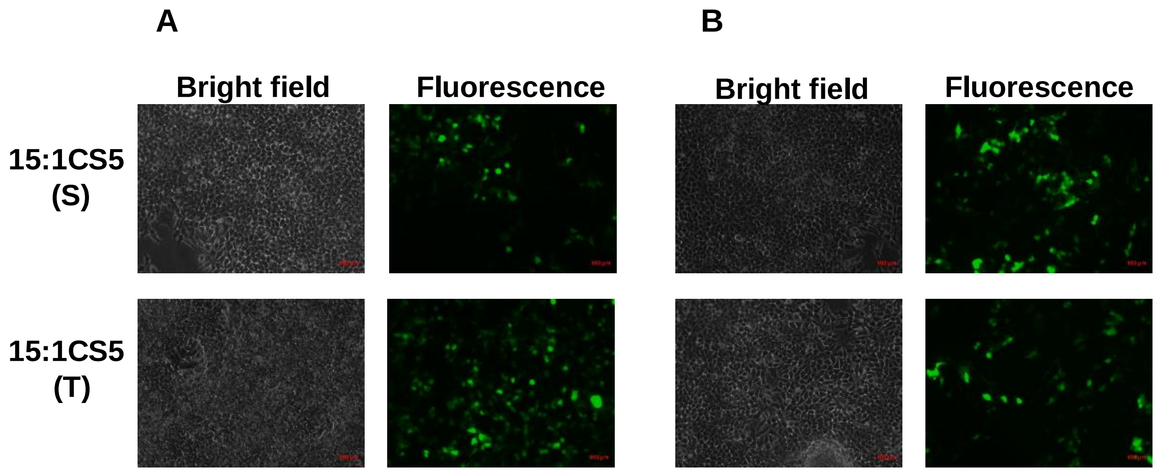

Figure S9 - **Representative images of fluorescence microscopy of transfected cells by 15:1CS5 (S) and 15:1CS5 (T) polyplexes.**
Transfection of 15:1CS5 (S) and 15:1CS5 (T) polyplexes with 100 µg of IGFBP-5, after 48h (A) and 72h (B). Amplification of 100X and scale bar represents 100 µm.

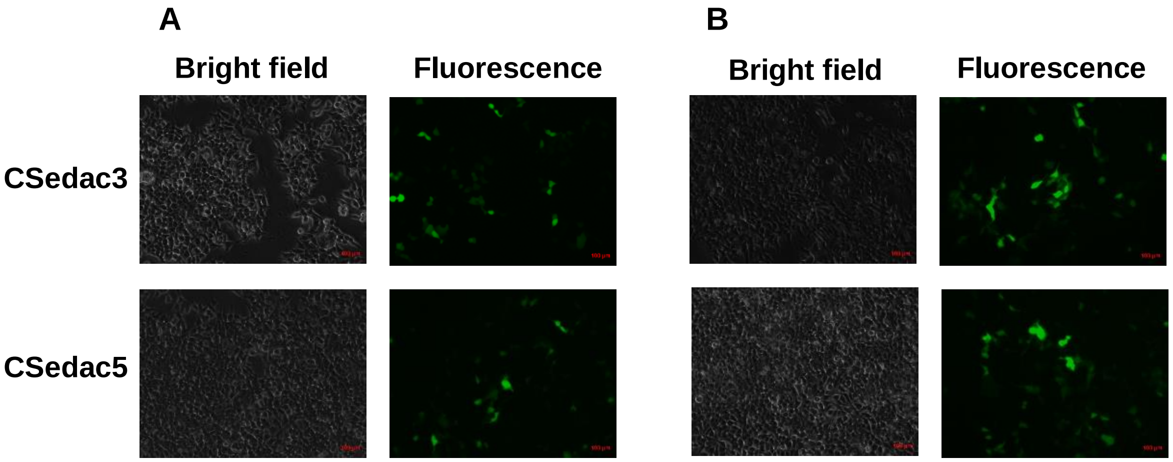

Figure S10 - **Representative images of fluorescence microscopy of transfected cells by CSedac3 and CSedac5 polyplexes.**

Transfection of CSedac3 and CSedac5 polyplexes with 100 µg of IGFBP-3 or -5, respectively. Cells were visualized after 48h and 72h, left and right panel, respectively. Amplification of 100X and scale bar represents 100 µm.
